# Thermochromic Silks for Temperature Management and Dynamic Textile Displays

**DOI:** 10.1007/s40820-021-00591-w

**Published:** 2021-02-14

**Authors:** Yang Wang, Jing Ren, Chao Ye, Ying Pei, Shengjie Ling

**Affiliations:** 1 School of Physical Science and Technology, ShanghaiTech University, 393 Middle Huaxia Road, Shanghai, 201210 People’s Republic of China; 2 School of Materials Science and Engineering, Zhengzhou University, Zhengzhou, People’s Republic of China

**Keywords:** Silk, Thermochromic, Smart textile, Dynamic display

## Abstract

**Highlights:**

- Wearable and smart textiles are constructed by integrating embroidery technology and 5G cloud communication, showing promising applications in temperature management and real-time dynamic textile displays.
- Thermochromism is introduced into the natural silk to produce high-performance thermochromic silks (TCSs) through a low cost, sustainable, efficient, and scalable strategy.
- The interfacial bonding of the continuously produced TCSs is in situ analyzed and improved through pre-solvent treatment and is confirmed using synchrotron Fourier transform infrared microspectroscopy.

**Abstract:**

Silks have various advantages compared with synthetic polymer fibers, such as sustainability, mechanical properties, luster, as well as air and humidity permeability. However, the functionalization of silks has not yet been fully developed. Functionalization techniques that retain or even improve the sustainability of silk production are required. To this end, a low-cost, effective, and scalable strategy to produce TCSs by integrating yarn-spinning and continuous dip coating technique is developed herein. TCSs with extremely long length (> 10 km), high mechanical performance (strength of 443.1 MPa, toughness of 56.0 MJ m^−3^, comparable with natural cocoon silk), and good interfacial bonding were developed. TCSs can be automatically woven into arbitrary fabrics, which feature super-hydrophobicity as well as rapid and programmable thermochromic responses with good cyclic performance: the response speed reached to one second and remained stable after hundreds of tests. Finally, applications of TCS fabrics in temperature management and dynamic textile displays are demonstrated, confirming their application potential in smart textiles, wearable devices, flexible displays, and human–machine interfaces. Moreover, combination of the fabrication and the demonstrated applications is expected to bridge the gap between lab research and industry and accelerate the commercialization of TCSs.
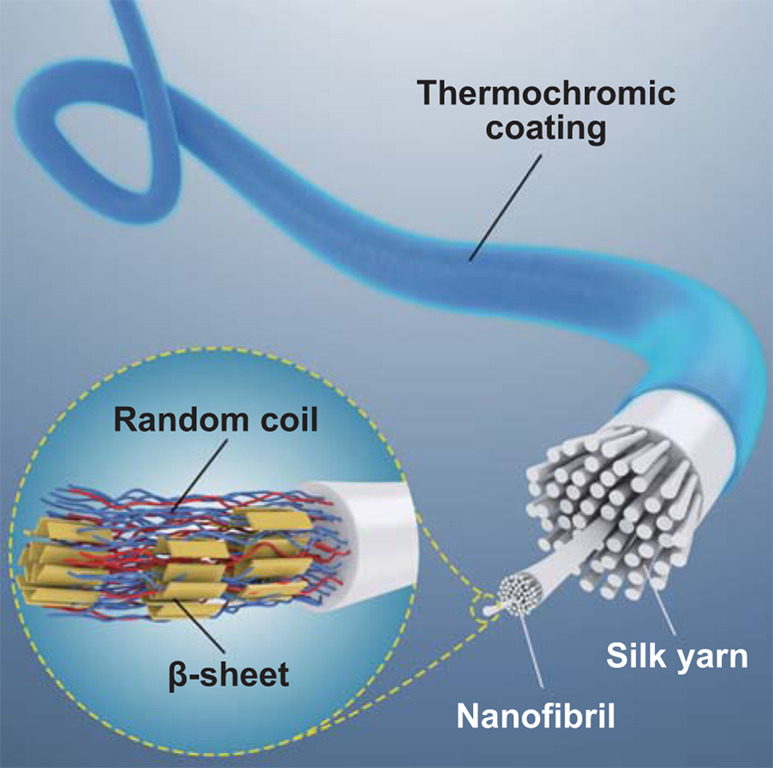

**Supplementary Information:**

The online version contains supplementary material available at 10.1007/s40820-021-00591-w.

## Introduction

Compared with synthetic polymer fibers, the unique *Bombyx mori* (*B. mori*) silk exhibits various benefits as a textile fiber [1–4]. Silk is lightweight yet strong and tough. It also has a natural sheen with high light absorbance, low static current generation, good resilience, excellent drape, and low heat conduction that makes it warm in the winter and cool in summer [5]. Direct functionalization of silk is expected to be a rational and effective strategy to develop high-performance smart textiles. To this end, different approaches have been studied, and feeding silkworm with functional components has been reported as the most direct way [6, 7]. Carbon nanotubes [8, 9], graphene [8, 10], silver nanoparticles [11], metal oxide nanoparticles [12–14], aggregation-induced emission nanoparticles [15], and organic dyestuffs [16–18] have been fed to the fifth instar silkworm toward producing silk cocoons with functional nanocomponents. However, the metabolic pathway of the silkworm larva is through the digestive system [19], which is a more or less straight tube leading from the mouth to the anus. The ingested substances, such as nanomaterials, are mainly excreted, and only a limited part can enter the blood circulation system and the silk gland due to the penetration restriction of the cytomembrane. In contrast to nanomaterials, the content of small molecule additives that can enter silk gland tissue is relatively high [16–18], but these small molecules are mainly distributed in the sericin layer, as silk fibroin (SF) is wrapped by three layers of sericin. Hence, most of these small molecules will be removed during the degumming process, and only some dyes, such as rhodamine, can be involved in the inner SF filaments.

Another functionalization approach is to dissolve silk into highly concentrated SF spinning dope followed by further spinning into regenerated silk fibers using industrial techniques, such as wet spinning and dry spinning [20]. The functional components are often added directly to the spinning solution. However, due to the spinnability requirements of the SF solution, the solid content of the silk protein needs to be more than 10% or even higher [21]. At such a high concentration, SF is accessible to gelation under external disturbances, so the total amount of functional components added is also greatly restricted. For example, when the amount of added carbon nanotubes is higher than 2% (relative to the mass of silk protein) [22, 23], the resultant composite solution will not be spinnable. In addition, when silk is processed by dissolution, dialysis, and spinning, the inherent structural and performance advantages of natural silk are destroyed [1, 2, 4], so the mechanical properties of many regenerated silk fibers are generally inferior to those of natural silks [20].

The current functionalization of silk mainly consists of two routes. (i) Electrical conductivity is imparted so that the silk can be used as an electrode or strain sensor to meet the needs of flexible and wearable devices [24]. To this goal, a series of methods were implemented, such as carbonization [25–27], dip-coating [28, 29], and solvent-based spinning [30]. (ii) It is relatively more difficult to color the silk, especially when a green and environmentally friendly approach is desired. Dyeing methods typically require harsh conditions involving toxic chemicals, high energy consumption, and pH changes as well as additional steps to restore the original properties of silk, such as chemical finishing, which results in large amounts of polluted wastewater. These operations significantly weaken the advantages of silk protein materials as sustainable materials. Genetic engineering techniques have been applied in the modification of silk proteins [31–37]. For example, a green fluorescent protein was gene targeted with the SF gene to produce green fluorescent silkworm cocoons. Bulk production of three colors (red, green, and orange) of fluorescent silks has also been obtained through transgenic silkworms [33]. However, the chromatographic range that can be generated by using the transgenic technique is still extremely limited, and the genetic stability of recombinant genes also needs further evaluation.

Therefore, our current work aims to develop a low-cost, effective, and scalable strategy to produce high-performance thermochromic silk (TCS), including formats of both fiber and yarn, and to further expand the applications of silks in thermochromic fields, as thermochromic textiles are widely desired in temperature sensors [38–40], smart windows [41], camouflage [42, 43], tactile logic [44], health monitoring [45], and photothermal therapy [46].

## Experimental

### In Situ Monitoring the Evolution of Thermochromic Ink on the Spinning Fiber

The *Bombyx mori* silkworm silk fibers (~ 50 μm in diameter, Fig. S1) are commercially available (Shengzhou Xiehe Silk Co., Ltd). The thermochromic inks and pigment powders were purchased from Zhongshan Jiahua Craft Printing Material Co., Ltd. Oily-based thermochromic inks containing polyvinyl chloride (PVC) resin (Foshan Ronggui Golden Globe Ink Co., Ltd), acetone (Shanghai Titan Scientific Co., Ltd) and thermochromic pigment powders, in which the mass percentage of PVC resin were 51.9%, 58.6%, 67.2%, 72.5%, 78.8%, and the mass percentage of thermochromic pigment powders was constant at 5%. A microfocus high-speed camera (i-SPEED 716, iX Camera, UK) was applied to visualize the evolution of thermochromic ink on the spinning fiber. Silk fiber was drawn out from the reservoir by a motor with a speed from 1 to 100 mm s^−1^ and coated with the thermochromic ink, then the evolution process on the fiber surface was recorded and analyzed. These processes were conducted in a chemical hood.

### Preparation of TCS Fibers and TCS Yarns

To achieve continuous spinning, an automated apparatus was designed, containing silk fiber feed, dip-coating tank and collection roller controlled by motor. During the spinning, silk fibers were first etched by HFIP (Shanghai Macklin Biochemical Co., Ltd), then passed through the coating tank with one end connected to the motor at a speed of 37.2 mm s^−1^. Time of silk fiber passing through the HFIP solvent, dye coating and drying is about 1.2, 7, and 21.5 s, respectively. All these processes were conducted in a chemical hood.

The TCS yarns were prepared by a self-designed continuous automatic twisting system. The system includes three main parts: (i) feeding shaft to provide TCS fibers, (ii) rotating device controlled by motor, and (iii) collecting roller controlled by motor. During twisting, four TCS fibers were used to prepare TCS yarns with the rotating and collecting speed of 258 rpm and 830 mm min^−1^, respectively. The obtained TCS yarns were weaved into different patterns on textiles using an automatic embroidery machine (MDP-S081C(200X300)S, Tajima Industries Ltd, Japan), and all the woven patterns were designed or derived from Tajima Writer Plus software (Tajima Industries Ltd, Japan).

### S-FTIR Microspectroscopic Measurements

The experiments were performed at BL01B in the Shanghai Synchrotron Radiation Facility (SSRF), Shanghai, China. TCS fibers were embedded in optimum cutting temperature compound and sectioned into slices of 5 μm by a cryostat (Leica CM1950, Germany). The TCS slice sample was measured by the Nicolet 6700 FTIR Spectrometer with Nicolet Continnum IR Microscope in transmission mode, and mapped spectroscopically in the mid-infrared range of 800–3800 cm^−1^ at a resolution of 8 cm^−1^ with 128 coadded scans and an aperture size of 20 × 20 μm^2^. All the spectral data were collected and analyzed by OMNIC9 software (Thermo Fisher Scientific, Waltham, MA, USA).

### Preparation of TCS/Carbon Fiber Yarns

A custom made co-wrapping spinning apparatus was used for fabricating TCS/carbon fiber yarns, which is composed of three key parts: (i) feeding motor to provide the core fibers, (ii) a co-wrapping system made by a rotator and a force controller, (iii) a motor-based collecting system. During the co-wrap spinning, the carbon fiber passed through the hollow tube at a speed of 42 mm min^−1^, and the speed of wrapping unit was fixed at 660 rpm to obtain full-packaged core-sheath yarns.

### Preparation of TCS-Based Fabrics for Multi-module Dynamic Display

The wearable and smart TCS-based fabrics were composited of digits knitted by TCS/carbon fiber yarns on the fabric and digital tube display drive equipment, which includes two main parts. The first part is the data transmission unit (DTU), which is responsible for receiving data from the 5G cloud platform via wireless network (Wi-Fi). The second part is the micro control unit (MCU), playing a function for processing data from DTU and driving the chip to display dynamic digits on the fabric. Besides, it also works for switching time and temperature display.

The TCS/carbon fiber yarn-based digits on the fabric were connected to digital tube display driving equipment by Dubond threads. After the equipment was connected to power and WIFI network, the time or temperature was dynamically displayed on the liquid crystal display (LCD) screen and fabric synchronously. And the yellow font on the LCD was the digit displayed on the fabric, and the switch between the time and the temperature display was realized by an external button.

### Mechanical Tests

TCS fibers were cut into segments with a length of 30 mm, then fixed on a hard-cardboard frame by using cyanoacrylate. After drying cyanoacrylate overnight, the hard-cardboard frame was mounted on the mechanical testing machine (Instron 5966 machine, Instron, Norwood, USA) and both side borders of the cardboard frame were cut away before the test so that the force was transmitted through the TCS fiber. Meanwhile, the initial length of the TCS fiber was measured with a caliper at zero load point, when the samples were tight and without any force exerted on it. Further, all of the tensile tests were carried at 25 °C and 50% relative humidity and applied with a constant tensile speed of 2 mm min^−1^ until they were broken. The cross-sectional area of the TCS fibers was calibrated by scanning electron microscope (SEM, Phenom Pro, China) before the tensile test and was estimated by ImageJ software (NIH).

### Sample Characterizations

The morphology of the TCS fibers, TCS/carbon fiber yarns and woven patterns were observed by high-resolution scanning electron microscope (SEM, JEOL JSM-7800F, Tokyo, Japan) at an acceleration voltage of 1 and 2 kV. Samples were coated with a 5 nm thick gold layer to provide conductivity before observation. The reflectance spectra of TCS fibers were examined in wavelength ranges (225–2500 nm) by an ultraviolet–visible-near infrared spectrometer (Agilent Cary 5000, USA).

### Derivation of the Evolution of Thermochromic Ink on the Spinning Fiber

The evolution can be simplified into two stages: i) the formation of a thin film (shell) layer, and ii) shrinkage of the continuous film layer into regular wave shape or even spaced droplets.

(i) When a fiber is drawn from the ink at a certain speed *U*, a film with a thickness of $e_{0}$ will be formed on the surface of fiber. In the region where the film forms, a flow takes place due to the Laplace pressure caused by the curvature of the film, which is written as:

1 $$p = \frac{\gamma }{{b + e_{0} }}$$

where $\gamma$ refers to surface tension of liquid, *b* and *e*_0 _refer to the diameter of fiber and thickness of the film, respectively.

During the drawing process, the pressure gradient along the dynamic meniscus is in balance with the viscous force, which can be written:

2 $$\frac{\eta U}{{e_{0}^{2} }}\sim\frac{\gamma }{{\lambda_{1} (b + e_{0} )}}$$

where $\lambda_{1}$ and *η* refer to dynamic meniscus length and liquid viscosity, respectively, and $U$ is the coating velocity. And the capillary number is determined as the ratio of viscous and capillary force, which is written as:

3 $$Ca = \frac{\eta U}{\gamma }$$

Among equation, $\lambda_{1}$ is unknown. In the LLD theory, it was proposed to match the static meniscus with the dynamic meniscus by balancing Laplace pressure to determine $\lambda_{1}$ [47]. As for the static meniscus, the Laplace pressure is zero when *b* is less than the capillary length ($b \ll a$). And the Laplace pressure in the dynamic meniscus is determined by the curvature of fiber and the second derivative of the meniscus profile [48]. Thus, the matching can be written as:

4 $$\frac{\gamma }{{b + e_{0} }} - \frac{{\gamma e_{0} }}{{\lambda_{1}^{2} }} \sim 0$$

5 $$\lambda_{1} \sim \sqrt {\left( {b + e_{0} } \right)e_{0} }$$

Thus, thickness of the film can be obtained by substituting Eq. 5 into Eq. 2 [49]:

6 $$e_{0} = \frac{{bCa^{{\frac{2}{3}}} }}{{1 - Ca^{{\frac{2}{3}}} }}$$

(ii) After coating the film on the surface of fiber, the film and fiber are moving together at a constant speed. Due to the Plateau-Rayleigh instability [50], the surface tension of the liquid will minimize the free surface area, resulting in instability. This annular film is unstable and can be gradually developed into a wave shape and even breaks into regularly spaced droplets. This instability is caused by the pressure in the film layer, including the hydrostatic pressure $\rho gR$ the Laplace pressure difference $\gamma /R$, where $R = b + e_{0}$. Due to the ratio of hydrostatic pressure and the Laplace pressure difference is $\frac{\rho gR}{\gamma /R} = \frac{{R^{2} }}{{a^{2} }}$, where the fiber diameter is ~ 50 μm. Because $R \ll a$, gravity can be ignored in the instability of the annular film. Thus, the instability is basically caused by the surface tension of the liquid. In this case, the critical conditions for preventing instability can be derived by calculating the energy difference between the annular liquid film and the corrugated film [49].

Assuming the liquid surface profile equation is written as:

7 $$e = e^{*} + \delta e\cos \left( {qx} \right)$$

where $e^{*} = e_{0} - \frac{{\delta e^{2} }}{4R}, q = \frac{2\pi }{{\lambda_{2} }}$, $\delta e \,{\text{and }}\,\lambda_{2}$ refer to amplitude and wavelength of the disturbance of oily-based ink, respectively.

Thus, surface energy between the modulated surface and the straight cylinder from which it originated (over a wavelength $\lambda_{2}$) can be derived as:

8 $$\Delta E = \mathop \int \limits_{0}^{{\lambda_{2} }} 2\pi \left( {b + e} \right)\gamma {\text{d}}s - 2\pi \left( {b + e_{0} } \right)\gamma \lambda_{2}$$

In the equation, corresponding liquid surface profile length (${\text{d}}s$) is written as:

9 $${\text{d}}s \approx {\text{d}}x\left[ {1 + \frac{1}{2}\left( {\frac{{{\text{d}}e}}{{{\text{d}}x}}} \right)^{2} } \right]$$

Then, ${\text{d}}s$ can be removed by substituting Eq. 9 into Eq. 8, we get

10 $$\Delta E = \frac{1}{4}\gamma \frac{{\delta e^{2} }}{b + e}2\pi \lambda_{2} \left( {q^{2} \left( {b + e_{0} } \right)^{2} - 1} \right)$$

Therefore, the critical conditions for preventing instability can be derived when the energy of the modulated surface is larger than the straight cylinder, hence the surface energy will increase and the perturbation is unfavorable, that is $\Delta E \ge 0$:

11 $$\lambda_{2} \le 2\pi \left( {b + e_{0} } \right)$$

For the film on the fiber ($e_{0} \ll {\text{b}}$), the wavelength is often $2\pi \sqrt 2 b$ [51], which is also the wavelength of the fastest growing model [49]. Therefore, when the wavelength $\lambda_{2}$ is equal to the circumference of the liquid column covering the fiber, the thickest film can be possible on the cylindrical fiber [52, 53]. Equation is written as:

12 $$2\pi \left( {b + e_{0} } \right) = 2\sqrt 2 b$$

13 $$e_{0\hbox{max} } = \left( {\sqrt 2 - 1} \right)b$$

Hence, we can obtain the critical condition for the fiber to be coated with a stable film, which can be written as:

14 $$e_{0} \le \left( {\sqrt 2 - 1} \right)b$$

### Color Analysis

The thermochromic evaluation of the inks and TCSs of various colors was recorded by a digital camera (Canon, EOS 80D) and the RGB data were analyzed and obtained by MatlabR2018b software (The MathWorks, Inc). According to CIE-XYZ and RGB conversion formula (Eqs. 15–18), we can obtain x and y values on CIE 1931 XY chromaticity diagram.

15 $${\text{gamma}}\left( x \right) = \left\{ {\begin{array}{*{20}l} {\left( {\frac{x + 0.055}{1.055}} \right)^{2.4} \left( {x > 0.04045} \right)} \hfill \\ {\frac{x}{12.92} \left( {x < 0.04045} \right) } \hfill \\ \end{array} } \right.$$

16 $$r = {\text{gamma}}\left( {\frac{R}{255}} \right), g = {\text{gamma}}\left( {\frac{G}{255}} \right), b = {\text{gamma}}\left( {\frac{B}{255}} \right)$$

17 $$\left| {\begin{array}{*{20}c} X \\ Y \\ Z \\ \end{array} } \right| = 100 \cdot \left| {\begin{array}{*{20}c} {0.4124} & {0.3576} & {0.1805} \\ {0.2126} & {0.7152} & {0.0722} \\ {0.0193} & {0.1192} & {0.9505} \\ \end{array} } \right| \cdot \left| {\begin{array}{*{20}c} r \\ g \\ b \\ \end{array} } \right|$$

18 $$x = \frac{X}{X + Y + Z} , y = \frac{Y}{X + Y + Z}$$

Under D65 illuminant conditions, conversion formula of CIEXYZ to CIE L*a*b* are written as [54]:

19 $${\text{L}}^{*} = \left\{ {\begin{array}{*{20}l} {116 \left( {\frac{Y}{100}} \right)^{{\frac{1}{3}}} - 16, } \hfill & {{\text{if}}\, \frac{Y}{100} > \left( {\frac{24}{116}} \right)^{3} } \hfill \\ {116 \left( {\frac{841Y}{10800}} \right), } \hfill & {{\text{if}}\, \frac{Y}{100} < \left( {\frac{24}{116}} \right)^{3} } \hfill \\ \end{array} } \right.$$

20 $$a^{*} = \left\{ {\begin{array}{*{20}l} {500 \left[ {\left( {\frac{X}{95.047}} \right)^{{\frac{1}{3}}} - \left( {\frac{Y}{100}} \right)^{{\frac{1}{3}}} } \right],} \hfill & { {\text{if}} \,\frac{Y}{100} > \left( {\frac{24}{116}} \right)^{3} } \hfill \\ {500 \left[ {\frac{841X}{10800} - \frac{841Y}{10800}} \right],} \hfill & { {\text{if}}\, \frac{Y}{100} < \left( {\frac{24}{116}} \right)^{3} } \hfill \\ \end{array} } \right.$$

21 $$b^{*} = \left\{ {\begin{array}{*{20}l} {200 \left[ {\left( {\frac{Y}{100}} \right)^{{\frac{1}{3}}} - \left( {\frac{Z}{108.8830}} \right)^{{\frac{1}{3}}} } \right],} \hfill & { {\text{if}}\, \frac{Y}{100} > \left( {\frac{24}{116}} \right)^{3} } \hfill \\ {200 \left[ {\frac{841Y}{10800} - \frac{841Z}{10800}} \right], } \hfill & {{\text{if}}\, \frac{Y}{100} < \left( {\frac{24}{116}} \right)^{3} } \hfill \\ \end{array} } \right.$$

## Results and Discussion

### Modulation of Colors and Evolutions of Thermochromic Inks

**Fig. 1:**
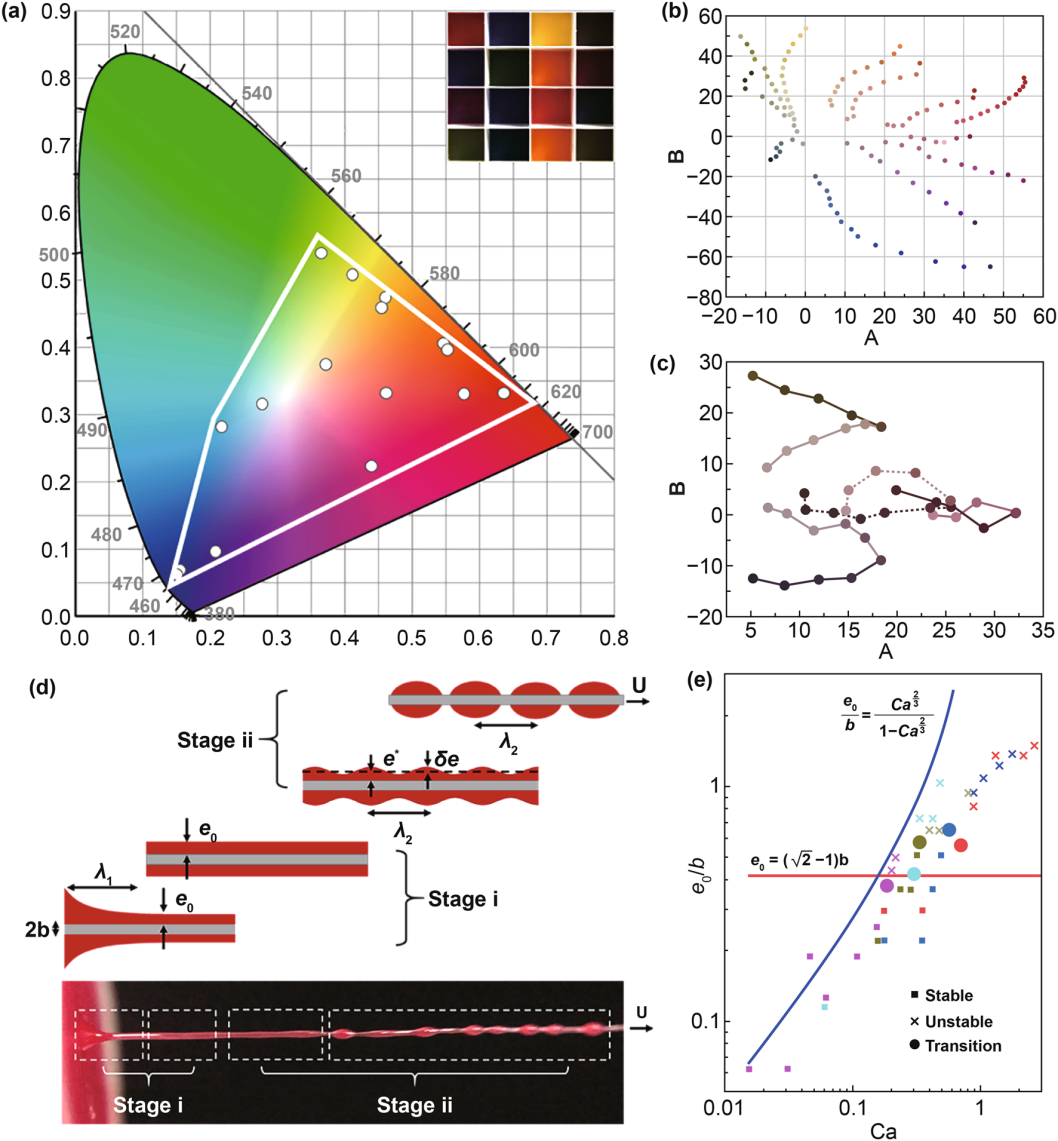
Modulation of colors and their evolutions of thermochromic inks as well as the morphology evolution of oily-Re-31 ink coating during yarn-coating-spinning. **a** CIE 1931 chromaticity diagram of modulated various colors by simply mixing ink of three elementary color (Re, Bl, Ye). The inset image is the ink of modulated various colors. **b**, **c** Two-dimensional chromaticity diagram of the color changes of different color inks obtained by two-color (**b**) and three-color (**c**) mixing method during heating process. Axis A goes from green (−A) to red (+A), axis B goes from blue (−B) to yellow (+B). **d** Schematic illustration and photograph of fiber being drawn from the ink at a certain speed U (Stage i) and the morphology evolution of liquid film on the fiber due to Plateau-Rayleigh instability (Stage ii). **e** Evolution of the relative film thickness on the fiber $\frac{{e_{0} }}{b}$ as a function of the capillary number Ca for different PVC inks (magenta, 51.9 wt%; cyan, 58.6 wt%; dark yellow, 67.2 wt%; blue, 72.5 wt%; and red, 78.8 wt%). States of transition, stable and unstable are displayed by symbols of different shapes. The blue continuous line according to theory represents the function $e_{0} = \frac{{bCa^{{\frac{2}{3}}} }}{{1 - Ca^{{\frac{2}{3}}} }}$, the red continuous line represents the function $e_{0} = \left( {\sqrt 2 - 1} \right)b$. (Color figure online)

Two specifically, low-cost, commercialized, and environmentally friendly thermochromic inks were first selected for silk functionalization, including aqueous-based (using aqueous solution as ink solvent) and oily-based (using polyvinyl chloride resin as ink solvent) systems. To make it easier to follow, the thermochromic inks were termed according to their properties, simplified as solvent-color-thermochromic temperature ink. For example, water-based red ink with a transition temperature of 34 °C is named as aq-Re-34 ink, where aq represents aqueous-based; similarly, oily represents oily-based. Starting from the three elementary color red (Re), yellow (Ye), and blue (Bl), we further modulated various color, such as orange, green, purple, cyan, and blue gray through two-color or three-color mixing so that TCS with more color gamut can have wider applications in different scenarios with less limitation for costume designing. As indicated in Fig. 1A, the color modulated by this simple mixing method can cover 36% of the color gamut on a CIE 1931 XY chromaticity diagram.

Through the temperature change process, the thermochromic behavior of the resultant system can be programmed. To better present the visual color change on a 2D plane (Fig. 1b), the CIELAB color space (also known as CIE L*a*b*) was applied to describe the thermochromic trajectories, as it approximates human vision better than the RGB and CMYK color models. CIELAB expresses color using three values: L* for lightness from black (0) to white (100), a* from green (–) to red (+), and b* from blue (–) to yellow (+). The CIELAB color space was designed so that the same amount of numerical change in these values corresponds to roughly the same amount of visually perceived change. By projecting the lightness onto the plane formed by a* and b*, this three-dimensional model can be depicted as a two-dimensional chromaticity diagram. As displayed in Fig. 1c, when the aq-Re-43, aq-Bl-43, and aq-Ye-43 inks were mixed in the ratio of 1:2:1, an aq-Bl,Gr-43 color ink was generated. Thereby, when the temperature was increased, the ink first changed its color to purple and then further to pale. Table 1 summarizes all the colors modulated through the mixing approach and their associated thermochromic evaluation.

**Table 1:** The colors that can be modulated through the mixing of the water-based red, blue, and yellow inks with a transition temperature of 43 °C and their associated thermochromic evaluation

Proportion (Re:Bl:Ye)	Resulting color	Thermochromic evaluation
1:1:0	Purple (Pu)	Pu → Pale (Pa)
0:1:1	Green (Gr)	Gr → Pa
1:0:1	Orange (Or)	Or → Pa
2:1:0	Magenta (Ma)	Ma → Pa
1:2:0	Violet (Vi)	Vi → Pa
2:0:1	Reddish Orange (Re,Or)	Re,Or → Pa
0:1:2	Lime (Li)	Li → Pa
0:2:1	Cyan (Cy)	Cy → Pa
1:0:2	Yellowish Orange (Ye,Or)	Ye,Or → Pa
1:1:1	Black Brown (Bl,Br)	Bl,Br → Re → Pa
2:1:1	Plum (Pl)	Pl → Re → Pa
1:2:1	Blue Gray (Bl,Gr)	Bl,Gr → Pu → Pa
1:1:2	Dark Yellow (Da,Ye)	Da,Ye → Br → Pa

Although considerable progress has been made in thermochromic fabrics, a tight adhesion of thermochromic materials to fabrics remains elusive. In most cases, an additional binder is applied, such as acrylate and polyurethane adhesive. However, adhesive usually increases the viscosity of the dyestuff composite too much, so only transfer printing or spray coating can be used for further processing. These techniques are generally only suitable for patterning macro-scale fabrics. Fiber and yarns, on the other hand, are more easily to be processed into different structural shapes to match the different application needs, and can also improve the coloring accuracy of the resulted fabric to the fiber or yarn scale, which is in the order of several hundred micrometers for silk fiber (Fig. S2). Therefore, a stable and uniform coating flow that warps on micro-scale fiber and yarns needs to be ensured during continuous coating process, while tight interfacial bonding between silks and inks needs to be achieved, as it determines the durability for a practical use.

### Morphology Evolution of Liquid Film During Ink Coating Process

Regarding the first issue, the stability and uniformity of the coating flow on fiber/yarns are different for different ink systems. In the actual experiments, a uniform coating was more easily achieved using aqueous-based inks due to the hydrophilicity of silk fiber (Fig. S3). However, when oily-based ink was used, both stable and unstable coating flow were observed. A microfocus high-speed camera was applied to visualize the evolution of oily-based thermochromic ink on the surface of the spinning fiber, which passed through the ink continuously at speed from 1 to 100 mm s^−1^. As presented in Fig. 1d, such an evolution can be simplified into two stages: (i) formation of a thin film (shell) layer, and (ii) shrinkage of the continuous film layer into regular wave-shaped or even spaced droplets. As a continuous and uniform ink coating is required, a theoretical model that relies on these experimental observations was first established to predict and screen the spinning parameters, including the viscosity *η* of the ink, surface tension *γ* of the ink, and spinning speed *U*. The synergistic effect of these spinning parameters can be represented by the capillary number *Ca* in Eq. 3 and according to the Landau–Levich–Derjaguin (LLD) theory, the thickness of the ink film layer $e_{0}$ formed in the continuous dynamic coating process can be directly predicted based on the value of *Ca* when *b* is known (Eq. 6). The detailed derivation of this equation is presented in the experimental section.

After a film layer is formed on the fiber surface, the film and fiber are moving forward together at a constant speed. Due to the Plateau-Rayleigh instability [50], surface tension of the liquid minimizes the free surface area, resulting in instability. This annular film layer is unstable and can be gradually developed into a wave shape and even break into regularly spaced droplets (stage ii in Fig. 1d, Movie S1). This instability is caused by the force in the film layer, including the hydrostatic pressure and the Laplace pressure difference. And the critical conditions for the fiber to prevent the occurrence of instability can be derived when surface energy of the modulated surface is larger than that of the straight cylinder ($\Delta E \ge 0$), hence the surface energy will increase and the perturbation is unfavorable, resulting in the final expression described in Eq. 14. The detailed derivation of Eq. 14 is presented in the experimental section.

In order to verify this model prediction, we prepared a series of inks with different physical properties (summarized in Table S1) and recorded their evolutionary behavior, while the silk fibers were spinning through them at different speeds (Fig. S4). As shown in Fig. 1e, the model accurately predicted the spinning conditions that were required to produce a uniform coating layer. When $\frac{{e_{0} }}{b} \le \left( {\sqrt 2 - 1} \right)$, the experimentally detected coating layer was indeed stable, and the critical transition from the liquid film to spaced droplets occurred when $\frac{{e_{0} }}{b}$ was in the range of 0.38–0.65 with an average value of 0.52, which is close to the theoretical value. Above this critical transition, the liquid film developed into droplets due to the Plateau-Rayleigh instability. In addition, when $Ca < 1$, the experimental $\frac{{e_{0} }}{b}$ values were also reasonably close to the theoretical values of $\frac{{Ca^{{\frac{2}{3}}} }}{{1 - Ca^{{\frac{2}{3}}} }}$. Therefore, Eq. 6 can be applied to predict the $e_{0}$ values. The deviation between the theoretical value and the experimental value only deviate from each other when $Ca > 1$, which is mainly due to the inertia of the reservoir standing still at large velocities. In fact, the situation of $Ca > 1$ is almost impossible due to the designed spinning process. Taking oily-Re-31 ink as an example, the spinning speed should exceed 300 mm s^−1^ if $Ca > 1$, corresponding to 37.2 mm s^−1^ in the actual experimental process.

On the basis of this theoretical guidance, a series of combinations of spinning parameters have been established for each ink to obtain TCS with a highly uniform coating layer, as summarized in Table S2, and the effectiveness of all these combinations has been confirmed by our screening experiments. For instance, using oily-Re-31 ink with a viscosity of 0.11 kg m^−1^ S^−1^ and surface tension of 0.033 kg S^−2^, TCS with a highly uniform coating layer can be obtained by continuously reeling with a speed of 37.2 mm s^−1^ for more than 72 h. Such spinning conditions can produce TCSs with length of ten kilometers, which are comparable with the continuous spinnable length of fibers without coating at the same reeling speed.

### Enhancing Interfacial Bonding Between Silks and Inks

The strong interfacial bonding between silks and inks is relatively more challenging to realize as most inks are hydrophobic, while silk is hydrophilic. Conventional industrial silk dyeing process usually requires cumbersome steps including heating (40–90 °C), soaking (10–50 min), repeated washing and dehydration, as well as chemical auxiliaries, which results in large amounts of polluted wastewater and energy consumption. On the other hand, thermochromic materials are more difficult to dye silk textiles due to their low reactivity. Researchers reported improved dyeing methods of creating chemical reactions between silk fibers and thermochromic pigments [55]. However, soaking and heating were still needed, while either silk fibers or regular pigments need to be modified.

**Fig. 2:**
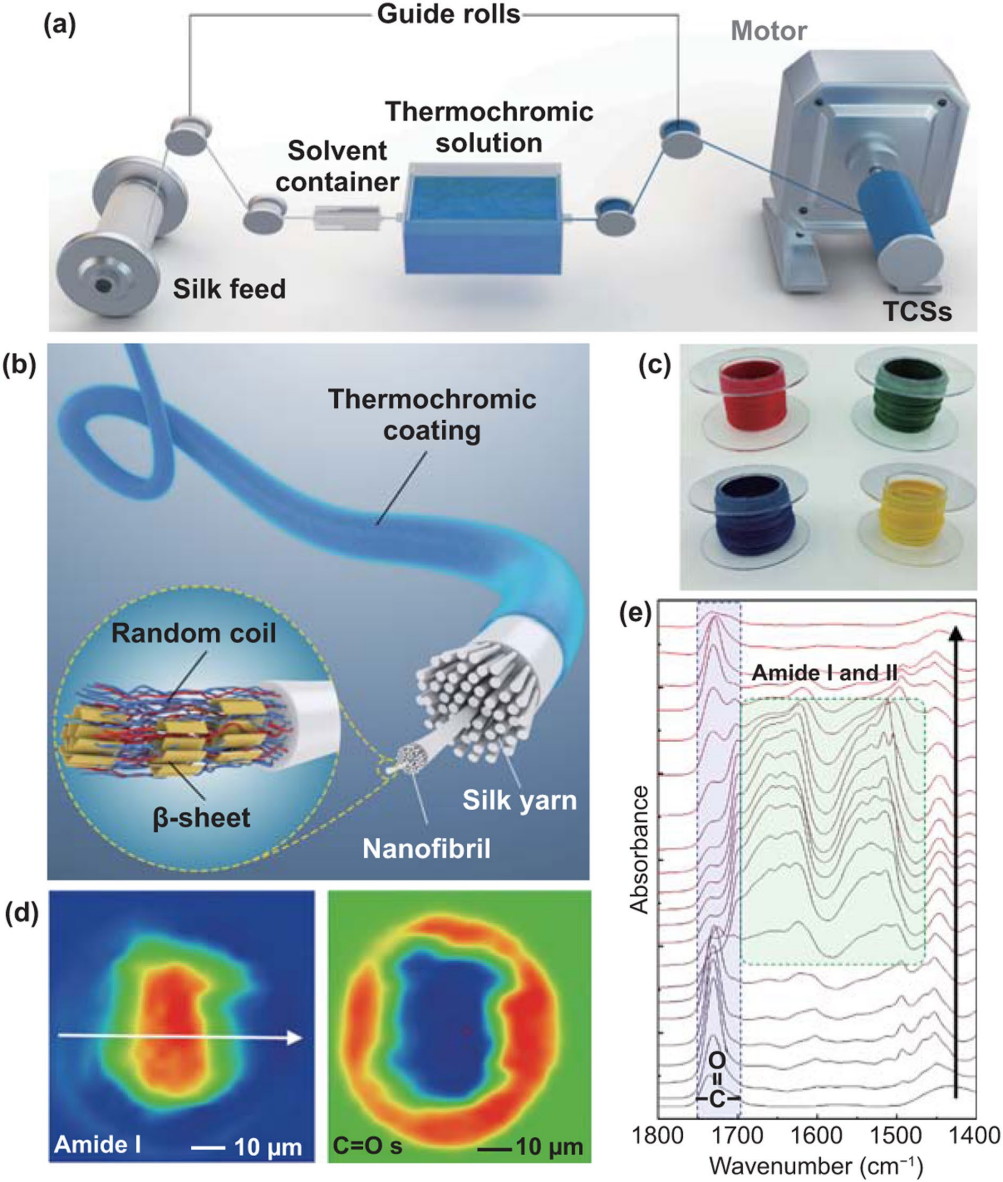
Spinning, structures, and properties of TCSs. **a** Schematic diagram of the continuous spinning device to produce TCSs. **b** Schematic illustration of the hierarchical interfaces in TCS fibers. **c** Photographs of red, green, blue and yellow TCS fibers. **d** S-FTIR spectroscopic images of TCS slice by integrating the characteristic peak of the silk at 1656 cm^−1^ (amide I) and the peak of inks at 1729 cm^−1^ (C=O stretching). **e** The single-pixel spectras extracted along the direction indicated by the white arrow in **d**

Our previous studies have found that formic acid and hexafluoroisopropanol (HFIP) can etch (partially dissolve) the silk fiber surfaces [28, 29, 56, 57]. The silk fiber then maintained its original configuration and mechanical property, while the fiber surface was only mildly dissolved. The dissolved silk fiber can further serve as a glue to bond with different types of composites, such as carbon nanotubes, inorganic nanoparticles and polymers. Therefore, a more efficient and pollution-free method to create a tight bonding between thermochromic inks and silk fibers can be achieved, without high temperature treatment and long-time soaking. Accordingly, an etching solvent container was installed before the coating tank (Fig. 2a). During the coating-spinning, a fiber/yarn was first passed through the etching solvent container, where the surfaces of the silk fibers are infiltrated and etched by the solvent to become sticky, which usually takes only 1.2 s. Therefore, as the silk fibers further pass through the coating tank, they can bind tightly with ink composites, while the hierarchical structure of the silk fibers is maintained (Fig. 2b). As a result, silk fibers with pre-coded colors were achieved and showed an even coating of the ink on the surface (Figs. 2c and S5). By stirring in water with soap for 48 h and rubbed with sandpaper for ten times, TCS fibers with HFIP pretreatment reveal improved interface stability and coating fastness compared with those without pretreatment (Figs. S6 and S7).

The surface coating and bonding between ink and silk was further imaged by S–FTIR. This technique can provide chemical information on a material with 5 μm spatial resolution [58–60]. By integrating the characteristic peak of the silk at 1656 cm^−1^ (amide I [58–60]) and the peak of inks at 1729 cm^−1^ (C=O stretching of the ester group [58–60]), the distribution of the ink around the silk was imaged (Fig. 2d). In agreement with the SEM results, the S–FTIR imaging of the TCS cross section shows that the ink layer was tightly covered around the silk core, while no defects was observed on the interface (Fig. 2d). The single-pixel spectra (Fig. 2e) extracted along the direction indicated by the white arrow in Fig. 2d also confirmed the abrupt change of the S–FTIR spectra at the interface, indicating that the characteristic spectrum of the ink converted into the specific absorption of silk protein without appearance of any void area.

### Mechanical Performance and Stability of TCSs

**Fig. 3:**
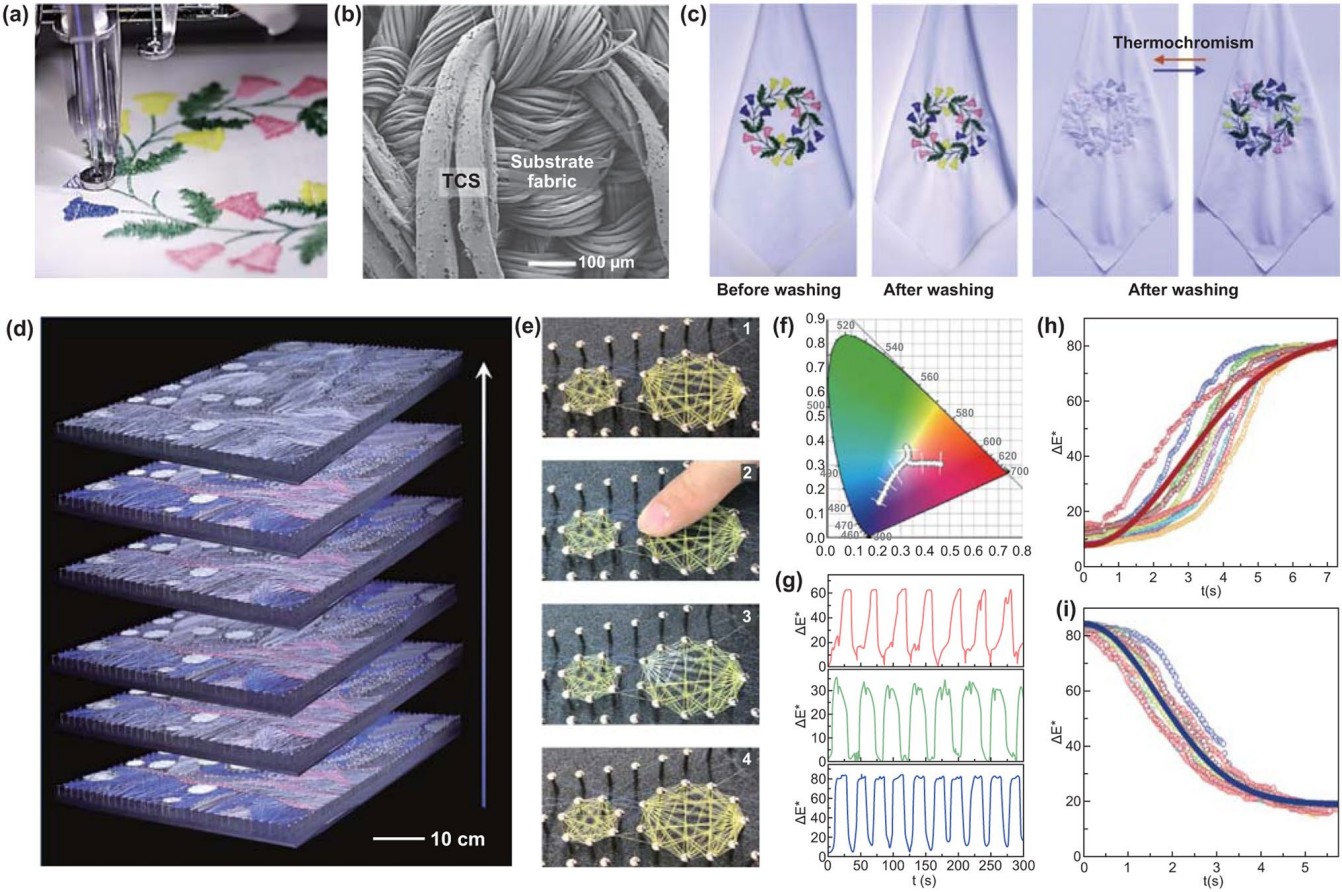
Washability and programmable thermochromism of TCSs. **a** Photograph of an automatic embroidery machine kintting the TCS yarns to a subtle logo pattern. **b** SEM image of the TCS yarns on the substrate fabric after machine washing. **c** Photographs of textile embroidery with color-changing pattern composed of TCS yarns before washing and after washing. **d** Photographs of color-changing TCS weaving pattern when the heat source passed from left to right. **e** Photographs of color-changing TCS weaving pattern approached by fingers. **f** Themochromic channel of red, green and blue TCS shown in CIE 1931 chromaticity diagram. **g** The variation of total color difference ΔE* of red, green and blue TCS with the temperature change controlled by the hot and cold circulating water. **h**, **i** Fitting plot of the variation of ΔE* when temperature rises (**h**) and drops (**i**)

As the structure of the silk was not damaged after these operations, the advantageous mechanical characteristics of silk were well retained in the resultant TCSs (Table S3 and Fig. S8). For example, the strength, toughness, and stiffness of the TCSs were 443.1 ± 44.2 MPa, 56.0 ± 8.5 MJ m^−3^, and 11.9 ± 1.2 GPa, respectively. These values are comparable to those of natural silk, which are 287.2 ± 58.8 MPa, 27.2 ± 6.6 MJ m^−3^, and 10.7 ± 2.7 GPa, respectively. Besides, after 50 cycles of stretching-releasing at 10% strain, the coating layer remains undamaged (Fig. S9). Furthermore, a TCS fiber knot also indicates the stable coating (Fig. S10). These mechanical merits support further processing by using automatic machines. As shown in Fig. 3a and Movie S2, the TCS yarns were directly woven into a subtle logo pattern using an automatic embroidery machine. SEM images of the TCS pattern revealed that the TCS yarns were intact and bond well with the substrate fabric (Fig. 3b). High-resolution SEM images (Fig. S11a) further displayed the full coverage of the yarn surface with ink, indicating that the sewing machinery did not destroy the bonding between the coating layer and the fiber surface.

The interfacial bonding between silks and inks was further confirmed by a series of washing tests. A TCS textile was washed by an automatic washing machine with soap (three repeated washing-dehydration process in one hour at room temperature), from one up to ten times, following by magnetic stirring for 48 h. The TCS patterns and their initial colors were well maintained due to the undamaged coating layer after washing (Figs. 3b and S11b). More importantly, compared with the original samples, the thermochromism function remained unchanged after washing, and no detectable change in sensitivity and brightness occurred (third and fourth images in Fig. 3c). The S–FTIR imaging of the TCS cross section further confirmed that after rinsing with deionized water, the distribution of the ink around the silk remained stable (Fig. S12).

### Programmable Thermochromism of TCSs

As illustrated in Fig. 3d and Movie S3, after the TCSs have been approached by the heat source, their color changed rapidly. Since each color has a different and specific response temperature, the color exhibits different response rates to heat sources. Among these colors, yellow is the fastest with a response rate, and red is the slowest, consistent with their thermochromic temperatures. In contrast, when the heat source was removed, the TCS pattern returned to its initial color. As the thermochromic temperature of yellow TCS is 28 °C, it could even detect the approach and departure of fingers (Fig. 3e). When the fingers approached, the corresponding position turned white, while it returned to yellow after the finger moved away. To quantitatively estimate the thermochromic response of each color, the TCSs were wound onto a heat pipe whose temperature can be precisely controlled by circulating water. The corresponding color changes were recorded and analyzed with an in situ video system. Here, Eq. 22 can be used to convert the three parameters L*, a*, and b* of the CIELAB color space into the Euclidean distance ΔE* as a single parameter

22 $$\Delta E^{*} = \left[ {\left( {\Delta L^{*} } \right)^{2} + \left( {\Delta a^{*} } \right)^{2} + \left( {\Delta b^{*} } \right)^{2} } \right]^{{\frac{1}{2}}}$$

where ΔE* represents the total color difference in sensation. It was found that the variation in ΔE* for all colors were synchronized with the temperature change (Fig. 3f, g). Therefore, such TCSs can be directly utilized to visualize temperature switches. Interestingly, all these ΔE* variations, both increases (Fig. 3h), and decreases (Fig. 3i), can be fitted by Gauss functions, where the ΔE* increase followed the following Eq. 23

23 $$\Delta E^{*} = 11.483.9 - \frac{524.6}{{5.5\sqrt {\frac{\pi }{2}} }} \cdot e^{{ - 2\left( {\frac{t - 0.067}{5.5}} \right)^{2} }}$$

and the ΔE* decrease followed Eq. 24

24 $$\Delta E^{*} = 18.92 + \frac{270.7}{{3.3\sqrt {\frac{\pi }{2}} }} \cdot e^{{ - 2\left( {\frac{t}{3.3}} \right)^{2} }}$$

### Temperature Management of TCS Fabrics

**Fig. 4:**
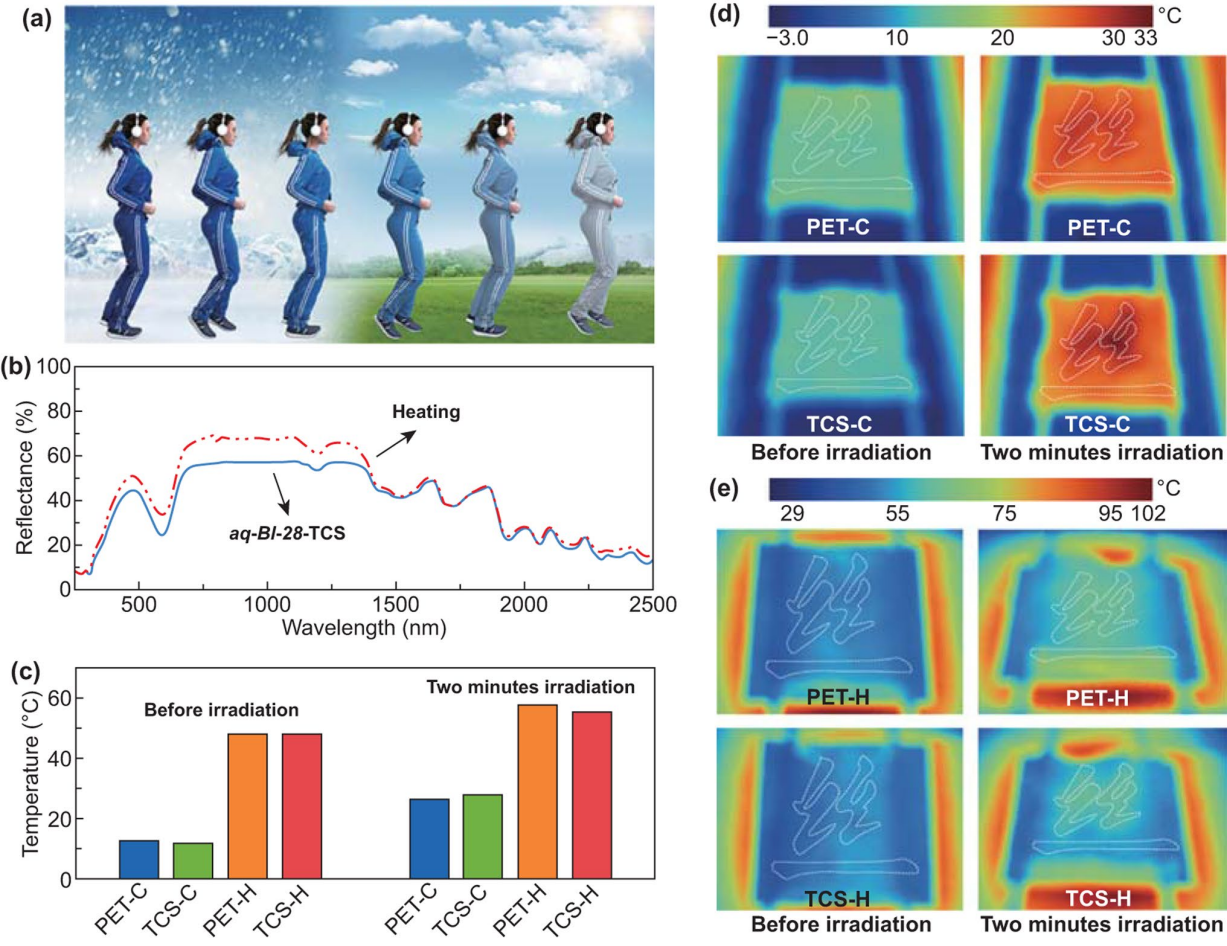
Temperature management of TCS fabrics. **a** Schematic showing thermochromic fabrics from cold to hot environment. **b** The reflectance spectrum of aq-Bl-28 TCS fibers before and after heating. **c** The temperature changes of PET and TCS Chinese “silk” characters on a polyester fabric before and after two minutes irradiation while the fabric was put into ice box or heating platform, a simulated cold (C) and hot (H) environment. **d**, **e** Thermal image of a polyester fabric with a PET and TCS Chinese “silk” character before and after two minutes irradiation at a simulated cold environment (**d**) and hot environment (**e**)

These advantages allowed TCSs to be applied in various scenarios. For example, in summer sports or outdoor work, the surrounding temperature is high enough to trigger the TCS textiles to light color (Fig. 4a). In this case, the degree of heat radiation received by wearing light-colored clothes is relatively low, and the wearer has a rather cool feeling. However, when the environmental temperature is low, wearing dark clothes would be warmer, as the dark color absorbs more heat. Here, thermochromic fabrics can be potentially utilized to realize a possible application of temperature management by controlling the dark and light color changes in the thermochromic behavior (Fig. 4b, c). The control of the heat reflectance by adjusting the light-and-dark color exchange of TCS fabrics under different environmental temperatures has been demonstrated (Fig. 4d, e). In this experiment, TCSs were embroidered on a polyester (PET) fabric to form the word “silk” in Chinese characters, while the PET “silk” character on PET fabric was used as the control sample (Fig. S13). The resultant fabrics were exposed to a simulated variable temperature environment. At low-environmental temperatures (Fig. 4d), the dark TCS fabric absorbed more heat than the PET base fabric and PET “silk” character after two minutes of irradiation, while at high-environmental temperatures, the temperature on light TCS “silk” was 27.9% lower than on the control PET “silk” after two minutes of irradiation (Fig. 4e).

### TCS-Based Dynamic Textile Displays

**Fig. 5:**
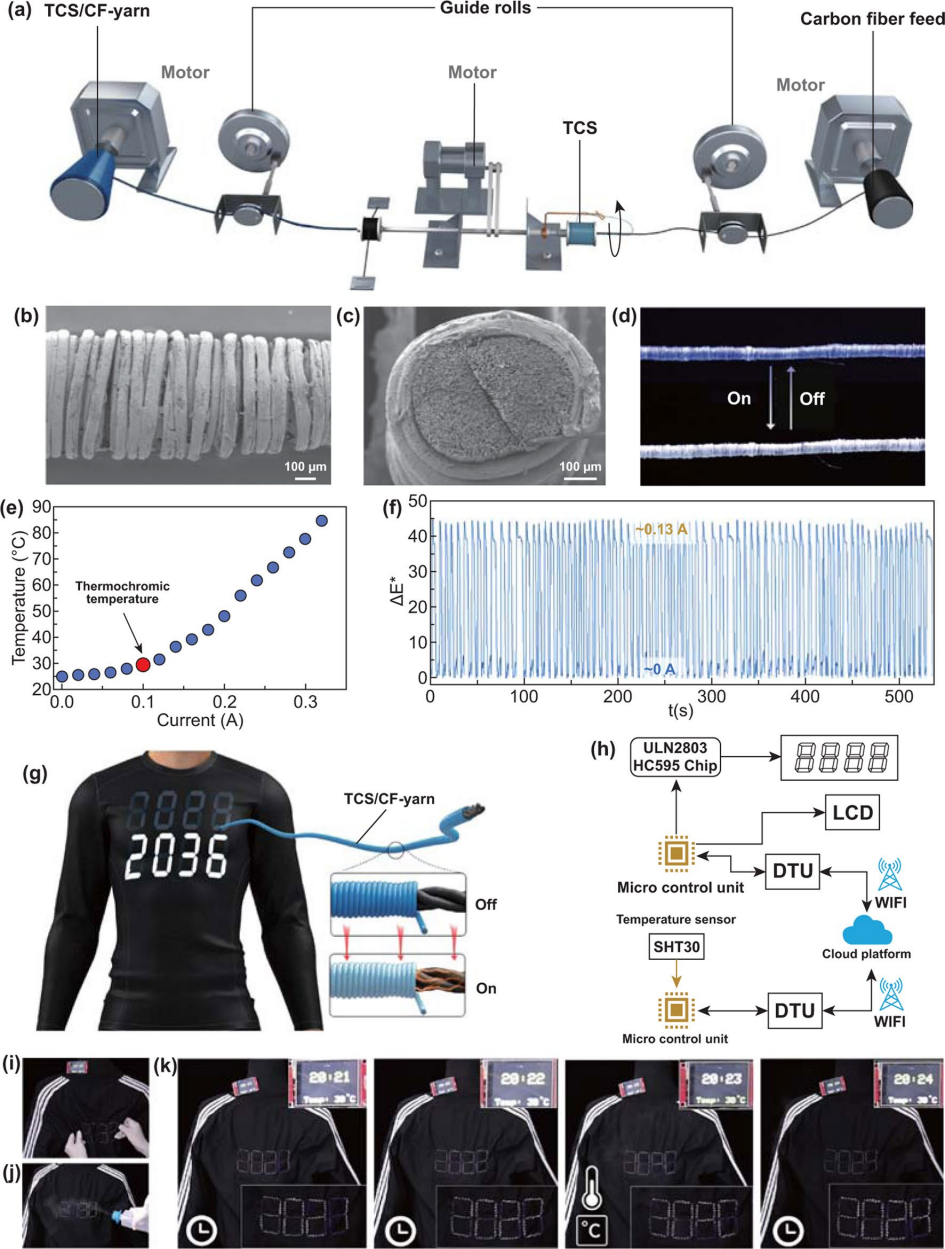
Design, fabrication and performance of TCS-based dynamic textile displays. **a** Schematic diagram of the co-wrap spinning apparatus for fabricating TCS/carbon fiber yarns. **b**, **c** SEM image of surface morphology (**b**) and cross section (**c**) of TCS/carbon fiber yarns. **d** Photograph of TCS/carbon fiber yarn showing smart color-changing effect by applying currents of 0.13/0 A. **e** Plot of temperature versus applied current on TCS/carbon fiber yarn, the red point refers to thermochromic temperature. **f** The stability of Euclidean distance, ΔE*, during repetitive power-on and power-off cycles by applying currents of 0.13/0 A. **g** Schemical illustration of a real-time digital display fabric constructed by TCS/carbon fiber yarns. **h** Schemical illustration of driving device for multi-module dynamic display, micro control unit (MCU) is responsible for switching time and temperature display, processing data from temperature sensor and driving the chip. The data transmission unit (DTU) plays a function for data transfer between MCU and cloud platform. **i**, **j** Real-time display under deformation (**i**) and rainning (**j**). **k** The multi-module dynamic textile display of switchable time and temperature

The application of TCS can be further extended to dynamic textile displays (Fig. 5). To this goal, we performed experiments where TCSs were continuously wrapped on carbon fibers by using a custom-made co-wrap spinning apparatus (Fig. 5a). During co-wrap spinning, the carbon fiber passed through the hollow tube at a constant speed, which was controlled by the traction motor. At the same time, the TCS fiber guided by the metal rotor rotated counterclockwise around the carbon fiber at the outlet of the hollow tube, thereby wrapping around the carbon fiber. According to the geometry of such a co-wrap spinning design, the ratio between the rotating speed $\omega$(rpm) of shell fibers and the drawing speed *v* (mm min^−1^) of core fibers (i.e., $\frac{\omega }{v}$) needs to match the critical condition of

25 $$\frac{\omega }{v} = \sqrt {\frac{1}{{D_{2}^{2} }} - \frac{1}{{4\pi^{2} \left( {r + D_{1} /2} \right)^{2} }}}$$

where $D_{1}$ and $D_{2}$ are the diameters of shell TCS fibers vertical and parallel to the composite yarn axis, respectively, and $r$ is the radius of core carbon fibers. When $\frac{\omega }{v} > \sqrt {\frac{1}{{D_{2}^{2} }} - \frac{1}{{4\pi^{2} \left( {r + D_{1} /2} \right)^{2} }}}$, the core fibers will be fully covered by the multilayer shell fibers. In contrast, when $\frac{\omega }{v} < \sqrt {\frac{1}{{D_{2}^{2} }} - \frac{1}{{4\pi^{2} \left( {r + D_{1} /2} \right)^{2} }}}$, the core fibers will be partially uncovered [61]. Here, the full-packaged core–shell structure is desired, as this configuration can completely appear in the color of the TCS, instead of a mixture of black and thermochromic colors. Accordingly, in practical spinning, the winding and drawing speeds were fixed at 660 rpm and 42 mm min^−1^, respectively. As shown in Fig. 5b, c, in the resultant core–shell yarns, the core carbon fiber was completely covered by TCSs.

In the TCS/carbon fiber yarns, the heat originates from the thermal resistance effect of carbon fibers in conducting electricity. Therefore, in order to trigger the thermochromism of the TCS/carbon fiber system, there is a relationship between current flowing through the carbon fiber core and its thermochromism performance; for example, the minimum current required for aq-Bl-31 ink is 0.103A (Fig. 5d, e). In this way, we can switch different color through the modulation of the current intensity of the carbon fiber. No obvious variation in the color switching rate was observed in a cyclic test (Fig. 5f). As this TCS/carbon fiber system is also automatically knittable, a real-time digital display fabric was constructed by embroidering the TCS/carbon fiber yarns onto a cloth and connect the circuit in the manner (Fig. 5g). Such a digital display fabric can directly display the numbers entered by mobile phones or computers (Fig. S14, Movie S4) and also display under certain extreme conditions, such as deformation, including stretching and tearing (Fig. 5i), as well as raining (Fig. 5j). In addition, if further introduced the remote weather data display module, such as atmospheric temperature, into the digital display fabric, a multi-module dynamic and real-time display can be achieved on a daily cloth (Fig. 5h). This design was very simple. A micro control unit (MCU) was responsible for processing data from temperature sensor and a data transmission unit (DTU) was used to transmit data to 5G cloud through Wi-Fi. Another DTU of the display driving device on the fabric was used to receive data, then the connected fabric and liquid crystal display (LCD) screen were simultaneously driven to display information. As shown in Fig. 5k and Movie S5, the environmental temperature and in-real time can be displayed on the cloth synchronously, and the time and temperature displays can be switched arbitrarily. Benefiting from high mechanical performance, the real-time display is expected to be applied to wearable devices and smart fabrics.

## Conclusions

In summary, we designed a synergistic strategy that combines yarn-spinning and continuous dip coating to produce TCSs with low cost, good sustainability, and high efficiency, thereby matching the potential scale-up requirements of smart textiles. The unique advantages of animal silk that compared with synthetic polymer fibers thus can be retained in this fiber/yarn functionalization system. The tensile strength and toughness of TCSs are 443.1 MPa and 56.0 MJ m^−3^, respectively, which are comparable to those of natural cocoon silk. These advantages allowed TCS to be automatically woven into arbitrary fabrics, and the possibility to be processed by industrial textile techniques. In addition, the pre-solvent treatment and coating integrated spinning strategy avoid subsequent highly polluting dyeing and finishing processes, which improved the sustainability of the animal silk production. In future work, the entire life cycle of animal silk fabrication and their applications will be very worthy of attention, because the factors influencing the sustainability of a material is not an individual process, but runs through from their birth to death [62, 63]. In this work, we realized the continuous preparation of 134-m-long yarns per hour at the laboratory scale, while all processing steps are carried out at room temperature and in a conventional and green solvent system. Besides, TCSs exhibited an elegant balance between mechanical properties and extensive thermochromic function, with a guaranteed tight and uniform surface bonding through careful experimental and theoretical analysis. Scale-up manufacturing will be a focus of our follow-up work, which is expected to make up for the gap between fundamental research and industrial production. At last, wearable temperature management and dynamic displays were demonstrated, realizing the true smart clothing and remote control. Therefore, TCSs are expected to have much broader application prospects as possible candidates for temperature sensors, smart windows, camouflage, tactile logic, health monitoring, and photothermal therapy.

## Supplementary Information

Supplementary material 1 (MP4 3580 kb)

Supplementary material 2 (MP4 6601 kb)

Supplementary material 3 (MP4 1973 kb)

Supplementary material 4 (MP4 11498 kb)

Supplementary material 5 (MP4 7243 kb)

Supplementary material 6 (PDF 972 kb)
