# Supplementary material for: Thermochromic Silks for Temperature Management and Dynamic Textile Displays
Source: Nanomicro Lett. 2021 Feb 14;13:72. doi: 10.1007/s40820-021-00591-w (PMC8187528; doi:10.1007/s40820-021-00591-w)
Supplement: Supplementary file 6 — Supplementary material 6 (PDF 972 kb) [file 40820_2021_591_MOESM6_ESM.pdf]

Supporting Information for

Thermochromic Silks for Temperature Management and Dynamic Textile Displays

Yang Wang[1, #], Jing Ren[1, #], Chao Ye[1], Ying Pei[2], Shengjie Ling[1, *]

[1]School of Physical Science and Technology, ShanghaiTech University, 393 Middle Huaxia Road, Shanghai 201210, P. R. China

[2] School of Materials Science and Engineering, Zhengzhou University, Zhengzhou, P. R. China

[#]Yang Wang and Jing Ren contributed equally to this work

*Corresponding author. E-mail: lingshj@shanghaitech.edu.cn (Shengjie Ling)

Supplementary Tables and Figures

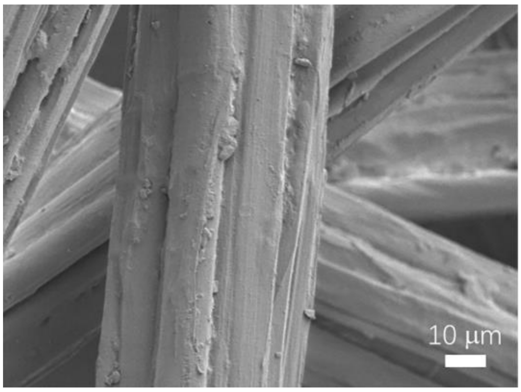

Fig. S1 SEM image of purchased silkworm fibers

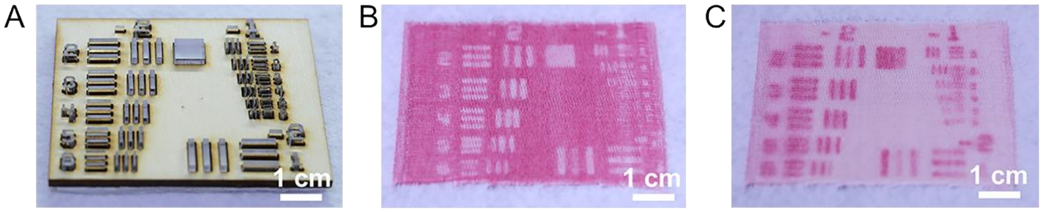

Fig. S2 Coloring accuracy of the TCS fabric. **A**) Photograph of a mold with the smallest accuracy of 0.5 mm used for the thermal and cold induction of the fabric. **B, C**) After the mold was heated or cooled, it was pressed on the fabric so that the high-temperature (**B**) and low-temperature (**C**) response areas appeared on the fabric

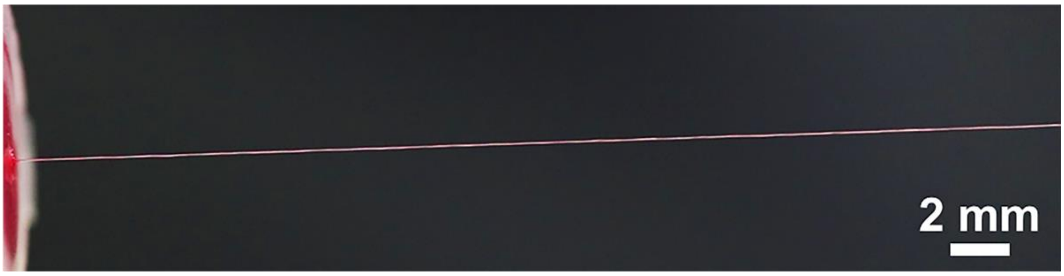

Fig. S3 Photograph of uniform coating formed on the silk fiber when using an aqueous-based ink

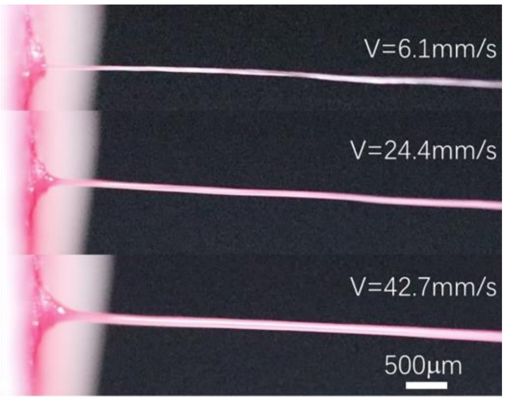

Fig. S4 Photographs of coating layer with different thickness formed on the silk fiber when using an oily-based ink (58.6 wt%) at different coating speed

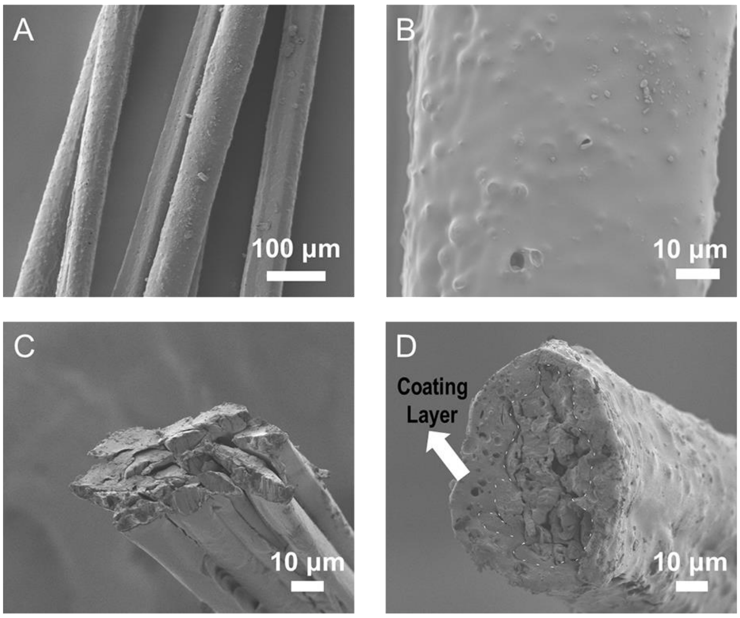

Fig. S5 Surface SEM images of TCS fibers (**A, B**) and cross-sectional SEM images of silk fiber (**C**) and TCS fiber (**D**). dots in **D** indicate the boundary between the coating layer and the core fibers

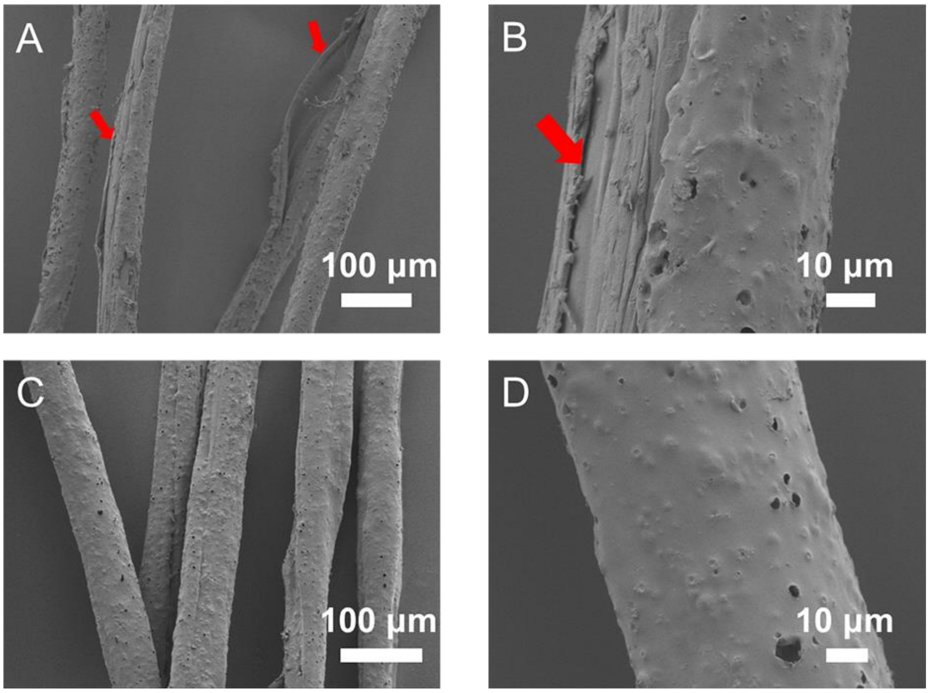

Fig. S6 SEM images of TCS fibers without (**A, B**) and with (**C, D**) HFIP pretreatment after stirred with soap for 48 hours. The red arrows indicate the surface damaged area

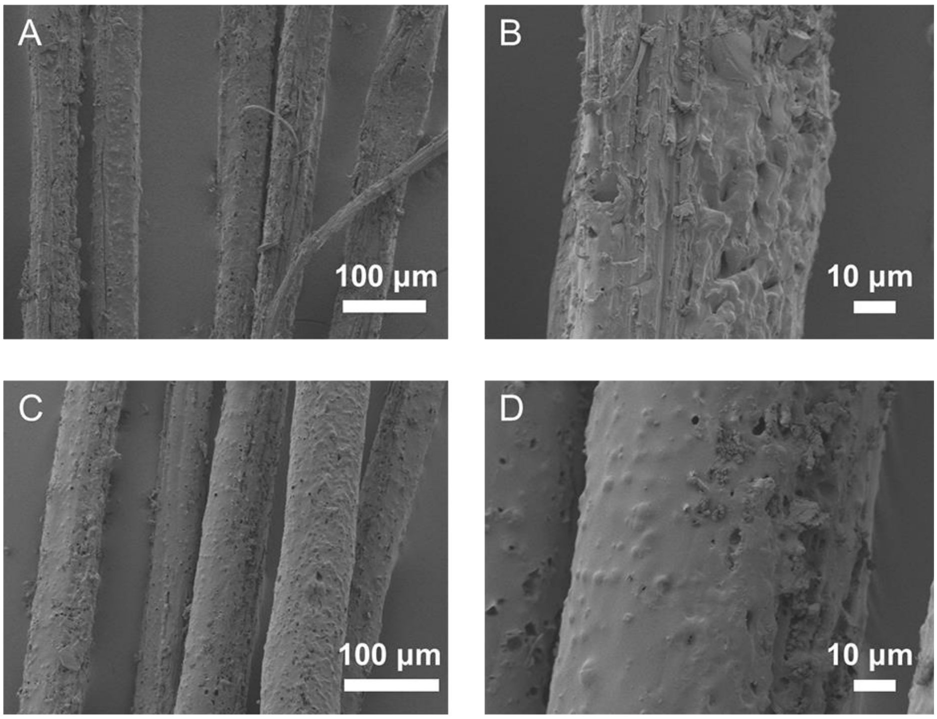

Fig. S7 SEM images of TCS fibers without (**A, B**) and with (**C, D**) HFIP pretreatment after rubbed with sandpaper for ten times

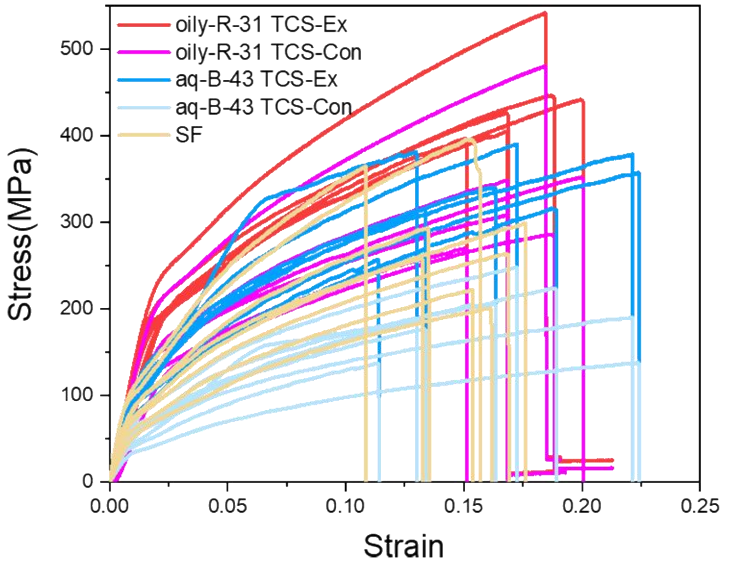

Fig. S8 Stress-strain curves of the natural silk fiber and TCS with a tensile speed of 2 mm min^{-1}. Oily-Re-31 TCS-Ex and TCS-Con refer to the TCS coating with oily-Re-31 ink. aq-Bl-43 TCS-Ex and TCS-Con refer to the TCS coating with aq-Bl-43 ink

-Ex means that the stress was calculated excluding the cross-sectional area of the coating layer; -Con means that the stress was calculated containing the cross-sectional area of the coating layer.

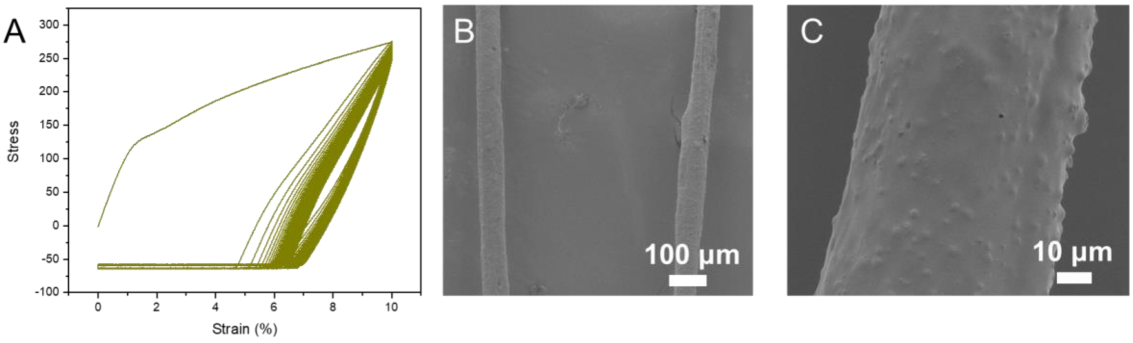

Fig. S9 Stress-strain curve of TCS with 50 cycles of stretching-releasing at a tensile speed of 2 mm min^{-1} (**A**) and SEM images of TCS fiber after 50 cycles of stretching-releasing (**B, C**)

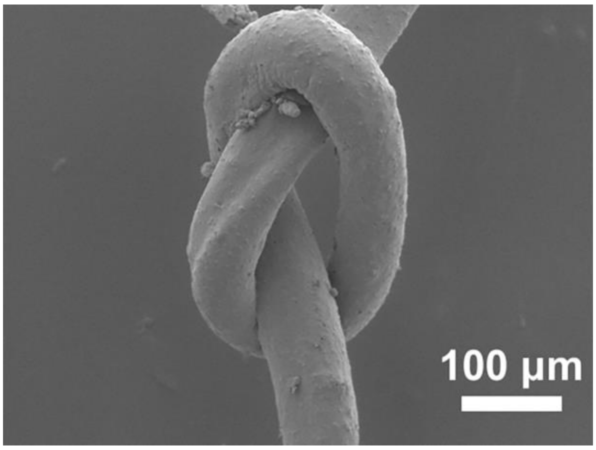

Fig. S10 SEM image of a TCS fiber knot

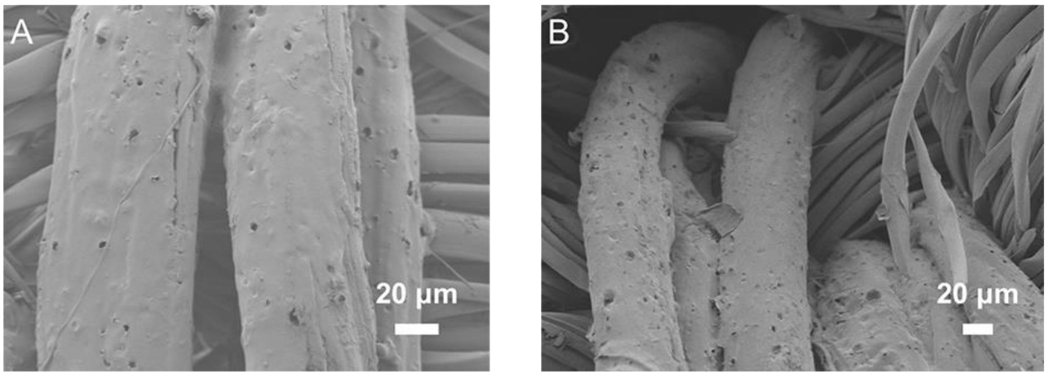

Fig. S11 High-resolution SEM image of TCS yarns on the substrate fabric after machine washing process for one time (**A**), and ten times and stirring with soapy water for another 48 hours (**B**)

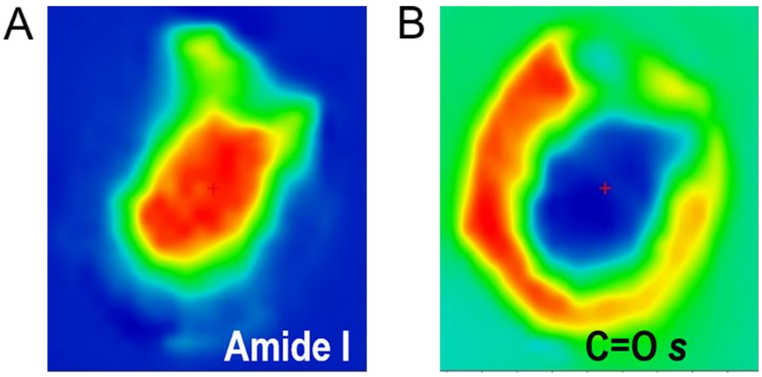

Fig. S12 S-FTIR spectroscopic images of TCS slice by integrating the characteristic peak of the silk at 1656 cm^{-1} (amide I, **A**) and the peak of inks at 1729 cm^{-1} (C=O stretching, **B**) after washing

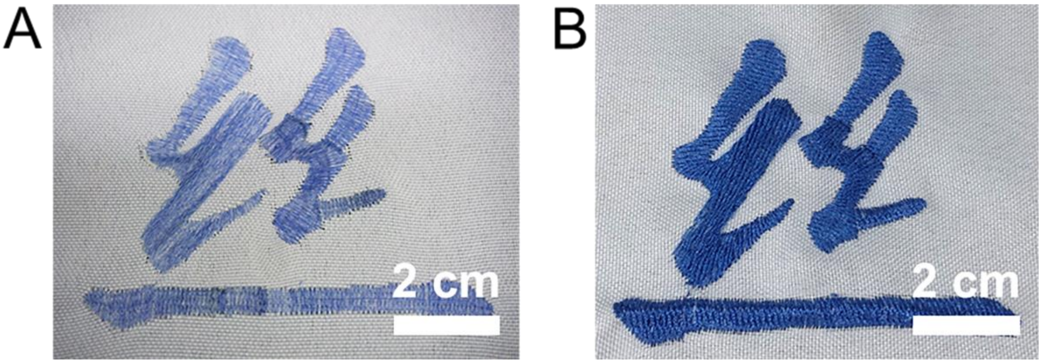

Fig. S13 Photographs of TCS (A) and PET (B) Chinese "silk" characters on PET fabrics

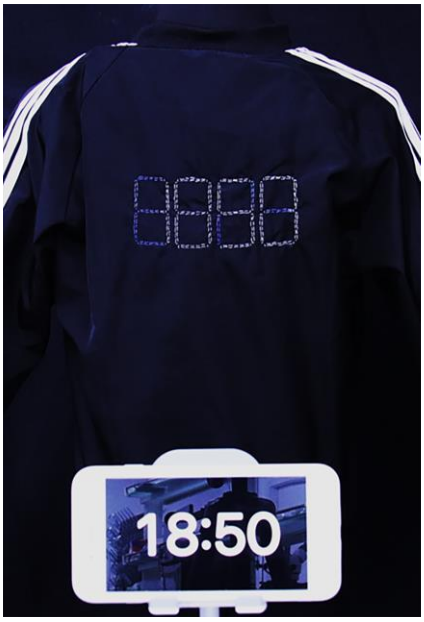

Fig. S14 Real display of digital fabric showing real time synchronized with mobile phone

Table S1 Physical properties of the PVC inks of different mass concentration (at 25°C)

Concentration (%)	ρ (Kg m^3)	η (Kg m^{-1} s^{-1})	γ (Kg s^{-2})	a (m)
51.9	948	0.11	0.0330	0.00189
58.6	981	0.3	0.0305	0.00178
67.2	992	0.762	0.0294	0.00174
72.5	1008	1.491	0.0259	0.00162
78.8	1029	4.465	0.0312	0.00176

Table S2 Different spinning parameters for PVC ink of different mass concentration to obtain TCS with a stable and unstable coating layer

Concentration (%)	U (mm s^{-1})	Ca	e_0/b	condition
	9.3	0.031	0.063	stable
51.9	37.2	0.124	0.188	stable
	60.5	0.201	0.438	unstable
	6.1	0.060	0.114	stable
58.6	24.4	0.240	0.421	stable
	42.7	0.420	0.728	unstable
	6.1	0.158	0.218	stable
67.2	12.2	0.317	0.506	stable
	18.3	0.475	0.649	unstable
	3.05	0.176	0.219	stable
72.5	9.76	0.563	0.651	stable
	15.25	0.879	0.938	unstable
	1.22	0.175	0.295	stable
78.8	4.27	0.559	0.611	stable
	6.1	0.872	0.824	unstable

Table S3 Comparison of mechanical properties of natural silk fibers and different TCS fibers

Materials	Strength (MPa)	Strain (%)	Modulus (GPa)	Toughness (MJ/m^3)
natural silk fiber	287.2 ± 58.8	14.8 ± 2.1	10.7 ± 2.7	27.2 ± 6.6
aq-Bl-43 TCS-1	187.8 ± 36.0	16.8 ± 3.9	6.2 ± 1.1	21.4 ± 7.1
aq-Bl-43 TCS-2	342.0 ± 40.0	16.8 ± 3.9	10.9 ± 0.6	39.3 ± 12.5
oily-Re-31 TCS-1	341.0 ± 64.3	17.6 ± 1.5	8.9 ± 1.4	43.2 ± 9.8
oily-Re-31 TCS-2	443.1 ± 44.2	17.6 ± 1.5	11.9 ± 1.2	56.0 ± 8.5
